# Perinatal Exposure to Glyphosate or a Commercial Formulation Alters Uterine Mechanistic Pathways Associated with Implantation Failure in Rats

**DOI:** 10.3390/toxics12080590

**Published:** 2024-08-14

**Authors:** Ailín Almirón, Virginia Lorenz, Jorgelina Varayoud, Milena Durando, María Mercedes Milesi

**Affiliations:** 1 Instituto de Salud y Ambiente del Litoral (ISAL), Facultad de Bioquímica y Ciencias Biológicas, Universidad Nacional del Litoral, Consejo Nacional de Investigaciones Científicas y Técnicas (CONICET), Santa Fe S3000, Argentina; aalmiron@fbcb.unl.edu.ar (A.A.);; 2 Cátedra de Fisiología Humana, Facultad de Bioquímica y Ciencias Biológicas, Universidad Nacional del Litoral, Santa Fe S3000, Argentina

**Keywords:** glyphosate, implantation failure, endometrial receptivity, proliferation, glandular function

## Abstract

Perinatal exposure to a glyphosate-based herbicide (GBH) or its active ingredient, glyphosate (Gly), has been demonstrated to increase implantation failure in rats. This study investigates potential mechanisms of action, analyzing uterine preparation towards the receptive state. Pregnant Wistar rats (F0) were treated orally with GBH or Gly (3.8 and 3.9 mg Gly/kg/day, respectively) from gestational day (GD) 9 until weaning. Adult F1 females became pregnant and uterine samples were collected on GD5 (preimplantation period). Histomorphological uterine parameters were assessed. Immunohistochemistry was applied to evaluate cell proliferation and protein expression of estrogen receptors (ERα and ERβ), cell cycle regulators (PTEN, cyclin G1, p27, and IGF1R-α), and the Wnt5a/β-catenin/FOXA2/Lif pathway. Both GBH and Gly females showed increased stromal proliferation, associated with a high expression of ERs. Dysregulation of PTEN and cyclin G1 was also observed in the Gly group. Reduced gland number was observed in both groups, along with decreased expression of Wnt5a/β-catenin/FOXA2/Lif pathway in the glandular epithelium. Overall, GBH and Gly perinatal exposure disrupted intrinsic uterine pathways involved in endometrial proliferation and glandular function, providing a plausible mechanism for glyphosate-induced implantation failure by compromising uterine receptivity. Similar effects between GBH and Gly suggest the active principle mainly drives the adverse outcomes.

## 1. Introduction

Embryo implantation is the first step to a successful pregnancy and failure in its progression is a major cause of infertility [1]. The implantation process is highly orchestrated and requires a synchronized and closed crosstalk between a competent blastocyst and a receptive uterus [2]. The time-limited window of implantation allows the endometrium to reach a state of adequate receptivity [3], which is characterized by morphological, functional, and molecular uterine changes regulated by the coordinated actions of the sex steroid hormones, 17β-estradiol (E2) and progesterone (P4) [2,3].

Several molecular pathways involving growth factors, cytokines, lipid mediators, adhesion molecules, and transcription factors, play a crucial role in orchestrating the dynamic changes that occur within the uterine endometrium [4,5,6]. A key endocrine pathway regulating the implantation process involves the interplay between, wingless-type MMTV integration site 5a (Wnt5a), β-catenin, forkhead box A2 (FOXA2), and leukemia inhibitory factor (Lif) [4,5,6]. Lif, a cytokine secreted by the uterine glands, is essential for embryo implantation, as it promotes uterine receptivity and facilitates blastocyst attachment and invasion [5]. Additionally, Wnt/β-catenin signaling interacts with and stabilizes FOXA2, a critical transcription factor for uterine function and fertility [6,7]. In the uterus, FOXA2 is mainly expressed in glands and is involved in the control of endometrial proliferation and decidualization [6]. It also acts upstream of Lif, regulating its expression and thereby modulating the receptive state of the endometrium [6]. Numerous knockout (KO) studies for Wnt5a, FOXA2, or Lif have reported endometrial glandular dysfunction and impaired stromal cell decidualization, demonstrating the importance of this endocrine pathway for successful implantation and fertility [6,8,9,10].

It is widely recognized that exposure to environmental compounds, such as pesticides, with endocrine-disrupting properties, may affect reproductive outcomes [11]. In this context, glyphosate (Gly), the active ingredient in glyphosate-based herbicides (GBH), has been postulated to exhibit eight out of ten key characteristics of an endocrine-disrupting chemical (EDC) [12]. Gly represents the most widely used broad-spectrum herbicide in the world, for pre- and post-emergent weed control, and as a desiccant in cereal crops [13]. Since the introduction of genetically modified glyphosate-resistant crops, the environmental prevalence of these herbicides has increased significantly over the past few decades [13]. Consequently, Gly and its main metabolite, aminomethylphosphonic acid (AMPA), have been identified in diverse environmental matrices, including water bodies [14,15], aquatic organisms [16], and indoor dust [17]. In addition, biomonitoring studies have detected the presence of Gly in urine [18] and serum [19] samples from pregnant women, as well as in breast milk [20].

The potential of Gly as an EDC has been at the center of debates. According to the United States Environmental Protection Agency (EPA), Gly is not classified as an EDC due to the lack of compelling evidence indicating its potential interaction with the estrogen, androgen, or thyroid pathways [21]. Similarly, the European Food Safety Authority (EFSA) asserts that pure Gly does not meet the criteria for EDC, even at doses that produce overt toxicity [22]. As a result, the use of Gly in Europe has been extended until 2034 [22]. Despite the conclusions of regulatory agencies, some studies have reported negative implications for human health. Indeed, urinary Gly levels in pregnant women were associated with preterm birth [23,24], longer anogenital distance at birth [24], and lower birth weight for gestational age [25], suggesting potential developmental effects associated with exposure during critical windows of human development.

In a previous study, we found that perinatal exposure to GBH or Gly induces impaired fertility in rats, as evidenced by increased implantation embryo losses [26]. We also demonstrated that implantation failure may be associated with hormonal and molecular changes that may prematurely close the implantation window and render the uterus refractory [26]. In the present study, we sought to expand and deepen our understanding of potential mechanisms involved in implantation failure by analyzing intrinsic uterine factors for proper endometrial receptivity. Our analyses included: (a) evaluation of uterine histomorphology; (b) quantification of cell proliferation and levels of expression of estrogen receptors (ERα and ERβ) and cell cycle regulators (PTEN, p27, cyclin G1, and IGF1R-α); (c) assessment of mediators of a key endocrine pathway for endometrial receptivity, such as Wnt5a/β-catenin/FOXA2/Lif pathway, during the preimplantation period.

## 2. Materials and Methods

### 2.1. Animals

All procedures were approved by the Institutional Ethics Committee of the Facultad de Bioquímica y Ciencias Biológicas (Universidad Nacional del Litoral, Santa Fe, Argentina) and were performed in accordance with the principles and procedures outlined in the Guide for the Care and Use of Laboratory Animals issued by the US National Academy of Sciences. In addition, the ARRIVE guidelines were followed. Animals were treated humanely and with care to minimize suffering. We used inbred Wistar-derived strain rats which were bred at the Instituto de Salud y Ambiente del Litoral (UNLCONICET) and housed in stainless steel cages with wood bedding under controlled environmental conditions of 22 ± 2 °C and a 14 h light/day cycle.

### 2.2. Chemicals

Gly (N-(phosphonomethyl) glycine) (CAS Number: 1071-83-6) of 96% purity was purchased from Sigma-Aldrich Inc. (St. Louis, MO, USA). The GBH used (MAGNUM-SUPER II) consisted of a water-soluble liquid formulation with 66.2% glyphosate potassium salt (equivalent to 54% *w*/*v* of glyphosate acid) as the active ingredient, plus undeclared coadjuvants and inert ingredients.

### 2.3. Experimental Design

Nulliparous mature female rats at the proestrus stage were housed overnight with males of proven fertility. The day on which sperm was identified in vaginal smears was designated as gestational day (GD) 1 [27]. Pregnant female rats (F0) were housed individually and randomly assigned to one of the three oral treatment groups using the Microsoft Excel random function = ()RAND. The three groups were as follows: (a) Control (*n* = 8), fed with a laboratory pellet chow-based paste; (b) GBH (*n* = 8), fed with a paste supplemented with a Gly commercial formulation; (c) Gly (*n* = 8), fed with a paste supplemented with Gly (active principle). The laboratory chow-based paste for each experimental group was prepared in accordance with the protocol outlined in our previous studies [28]. Briefly, an optimized amount of pellet (Nutrición Animal, Santa Fe, Argentina) and water were mixed. For the GBH and Gly groups, the standardized quantities of a commercial formulation or Gly, respectively, were added to the mixture to achieve doses similar to those observed in our previous studies in which we detected implantation failures [26,28]. F0 dam’s body weight and food intake were recorded three times per week throughout the treatment to calculate the actual Gly dose [26]. The doses were 3.8 and 3.9 mg of Gly/kg bw/day for the GBH and Gly groups, respectively. These doses are relevant because they are in the order of magnitude of the reference dose (RfD) for Gly, as indicated by the Environmental Protection Agency [29] based on developmental toxicity studies.

It is noteworthy that in a previous study [26] using the same doses as in the present work, serum glyphosate levels in F0 dams (0.037 and 0.013 mg/L in GBH and Gly groups, respectively) were in the order of magnitude as the mean concentration detected in serum samples from pregnant farmers (0.0175 mg/L) [19] and the highest concentrations documented in the plasma of non-occupationally exposed men (0.0141 mg/L) [30]. Consequently, the levels of circulating Gly in rats achieved by applying our experimental model are of relevance when compared to those found in peripheral blood in humans.

F0 dams received oral treatment from GD9 (after embryo implantation) until the weaning period (postnatal day (PND) 21). Following the delivery of the F1 pups, they were weighed and sexed according to the anogenital distance. A total of eight pups per litter, preferably four males and four females, were left with F0 lactating dams. No signs of maternal toxicity or significant differences in weight gain or food intake between groups were observed during the experiment. No changes in gestational length, maternal care, litter size, and pup sex ratio were observed among the experimental groups [26].

At PND21, one female offspring from each F0 dam was randomly selected to avoid potential litter effects. From PND21 until the conclusion of the experiment, all animals were provided with ad libitum access to tap water and pellet chow (without the addition of Gly or GBH). After PND90, vaginal smears were obtained every morning and F1 female rats (*n* = 8/treatment) at the proestrus stage were housed individually with untreated males of proven fertility. The presence of spermatozoa in vaginal smears was recorded as GD1. No changes in mating periods or pregnancy rates were observed between the experimental groups. To evaluate whether GBH or Gly could affect key events in the preparation of the uterus for the receptive state, animals were euthanized on the morning of GD5. This time corresponds to the late preimplantation period; since in our colony, implantation occurs on the evening of GD5. Uterine samples were collected, fixed in 10% formalin buffer for 6 h at 4 °C, embedded in paraffin, and processed for histology and immunohistochemistry.

### 2.4. Histomorphological Analysis

The histomorphological analysis was conducted on longitudinal sections of the uterus (5 μm thick) stained with Mayer’s hematoxylin and eosin under a light microscope (Olympus BH2 microscope; Olympus, Tokyo, Japan). The number of glands per field was calculated by counting the number of glands in 10 randomly selected fields using a Dplan 20× objective. Luminal epithelial cell height (LECH) was measured from the apical surface to the basement membrane in areas devoid of folds, as previously described [31]. The thickness of the subepithelial stroma and myometrium was analyzed using ImageJ 1.54F software (NIH, Bethesda, MD, USA; imagej.net/ij/index.html, accessed on 7 August 2024). For the LECH, subepithelial stroma, and myometrial layers, at least 10 fields were recorded in each section using a Dplan 40× objective (numerical aperture = 0.65; Olympus). Analyses were performed in three uterine sections per animal, with a 50-μm separation between sections.

### 2.5. Immunohistochemistry Assays

Immunohistochemistry was performed to determine endometrial proliferation by assessing the Ki67 marker and the protein expression of ERα, ERβ, Wnt5a, β-catenin, FOXA2, Lif, and the cell cycle regulators, PTEN, cyclin G1, p27, and IGF1R-α. Briefly, uterine longitudinal sections (5 μm thick) were deparaffinized and rehydrated in graded ethanol, and a microwave pretreatment was applied for antigen retrieval. Endogenous peroxidase activity and non-specific binding sites were blocked, and samples were incubated with the specific primary antibody (overnight at 4 °C) at the dilutions indicated in Table 1. Following a 30-minute incubation with biotin-conjugated secondary antibodies (Table 1), reactions were developed using the avidin-biotin-peroxidase method with diaminobenzidine (DAB) (Sigma-Aldrich) as a chromogen substrate. Each immunohistochemical run included positive and negative controls. No positive or background signal was detected in the negative control for any of the proteins under study. For Ki67 quantification, the samples were counterstained with Mayer’s hematoxylin (Biopur, Rosario, Argentina).

### 2.6. Quantification of Cell Proliferation and Protein Expression

Uterine samples were evaluated using an Olympus BH2 microscope with the Dplan 40× objective for cell proliferation and the Dplan 20× objective for protein expression. A point grid was employed to obtain the proliferation index, which was expressed as the volume fraction (*Vv*) of the positive cells. This was calculated as follows: *Vv = Pi/P*, where *Vv* is the estimated volume fraction of the object, *Pi* is the number of incident points over the positive cells for Ki67, and *P* is the total number of incident points over the area examined [35]. A minimum of 30 randomly selected fields per section (50 μm apart) and three sections per animal were analyzed. The uterine expression of ERα, ERβ, Wnt5a, β-catenin, FOXA2, Lif, PTEN, cyclin G1, p27, and IGF1R-α was evaluated by image analysis using the ImageJ 1.54F software (NIH, Bethesda, MD, USA; https://imagej.net/ij/index.html, accessed on 7 August 2024). Images were recorded using a Spot Insight V3.5 color video camera attached to the microscope (Olympus BH2). The results were expressed as integrated optical density (IOD), a dimensionless parameter that represents the average intensity of the positive cells and the relative area occupied by those cells [36]. The expression of PTEN, cyclin G1, p27, and IGF1R-α was quantified in the subepithelial stroma (a 200-μm-wide area adjacent to the epithelium). The expression of Wnt5a, β-catenin, FOXA2, and Lif, was quantified in the glandular epithelium. The quantification for ERα and ERβ expression was conducted in both the subepithelial stroma and the glandular epithelium. To quantify the protein expression, at least 10 randomly selected fields per section (50 μm apart), and three sections per animal were analyzed.

### 2.7. Dual Immunofluorescence Staining

A dual immunofluorescence staining procedure was applied to assess the colocalization of Wnt5a/β-catenin and FOXA2/Lif in the uterine glandular epithelium. In brief, the uterine sections were subjected to microwave for antigen retrieval after deparaffinization and rehydration. The sections were then blocked with sodium borohydride for 40 min to reduce autofluorescence and with normal horse serum for 1 h to minimize nonspecific background. Samples were incubated with primary antibodies (Table 1) overnight at 4 °C. The secondary antibodies (Table 1) were incubated for 1 h, after which the sections were washed in three changes of PBS for a total of 45 min. The cell nuclei were stained with 4′6-diamidino-2-phenylindole dihydrochloride (DAPI) (Fluka; Sigma). Samples were mounted in Prolong Gold fluorescent mounting medium (Invitrogen) and stored in the dark at room temperature. Negative controls were incubated with nonimmune serum. Analyses were performed using a Leica TCS SP8 confocal microscope (Laser 405, 488, 514, 552, 638 mm).

### 2.8. Statistical Analysis

All results are shown as the mean ± SEM. Data were analyzed using a Kruskal-Wallis test, followed by Dunn’s method for multiple comparisons. Statistical analyses were carried out using GraphPad Prism Version 5.03 software (GraphPad Software, San Diego, CA, USA). *p*-values lower than 0.05 were considered significant.

## 3. Results

### 3.1. Uterine Histomorphology on GD5

**Figure 1:**
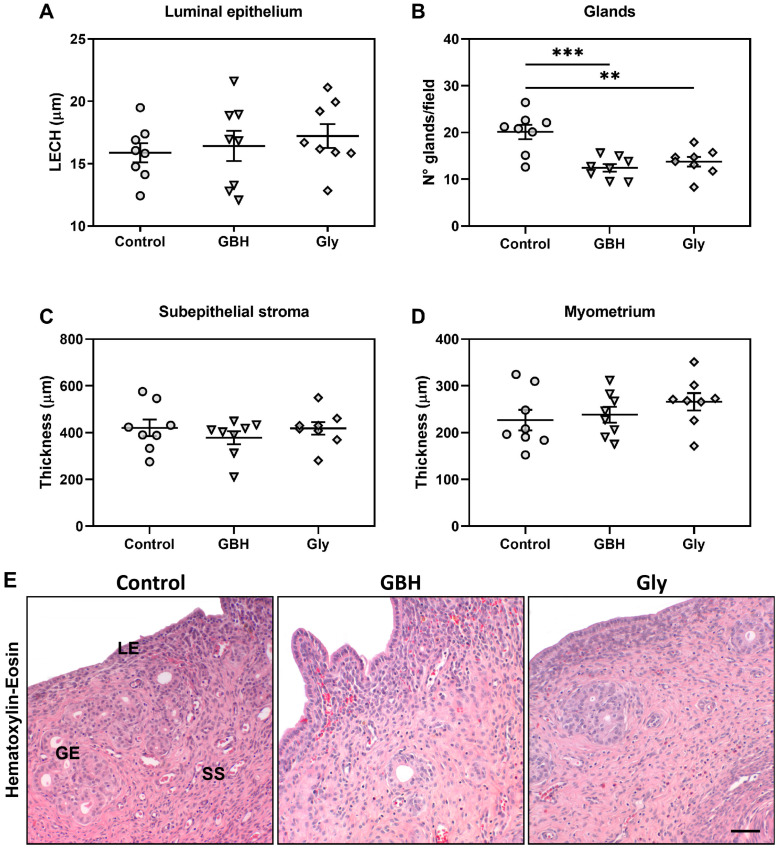
Effects of perinatal exposure to GBH or Gly on uterine morphology on GD5. (**A**) luminal epithelial cell height (LECH), (**B**) Number of endometrial glands per field, (**C**) thickness of the subepithelial stroma, and (**D**) thickness of the myometrium. Dots represent individual measures and solid lines represent the mean ± SEM of 8 animals per group. Asterisk denotes statistical significance (** *p* < 0.01, *** *p* < 0.001). (**E**) Representative photomicrographs of uterine sections stained with hematoxylin-eosin show reduced number of glands per field. LE: luminal epithelium; SS: subepithelial stroma; GE: glandular epithelium. Scale bar: 100 µm.

There were no differences in the LECH (Figure 1A) or in the thickness of the subepithelial stroma (Figure 1C) and myometrium (Figure 1D) between groups. However, a significant decrease in the number of glands per field was observed in the GBH- and Gly-exposed females in relation to control ones (Figure 1B,E) (*** *p* < 0.001 Control vs. GBH; ** *p* < 0.01 Control vs. Gly).

### 3.2. Stromal Cell Proliferation and Protein Expression of Estrogen Receptors and Cell Cycle Regulators

**Figure 2:**
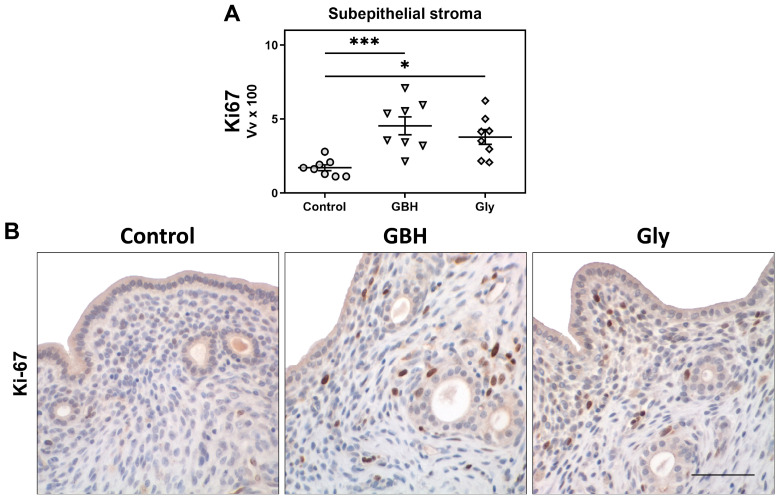
Effect of perinatal exposure to GBH or Gly on stromal cell proliferation on GD5. (**A**) Stromal cell proliferation was measured using Ki67 immunodetection and expressed as volume fractions (Vvx100). Dots represent individual measures and solid lines represent the mean ± SEM of 8 animals per group. Asterisk denotes statistical significance (* *p* < 0.05; *** *p* < 0.001). (**B**) Representative photomicrographs of Ki67 immunodetection in stromal cells. Scale bar: 100 µm.

Both GBH- and Gly-exposed females showed an increase in cell proliferation in the subepithelial stroma compared to the control group (*** *p* < 0.001 vs. GBH, * *p* < 0.05 vs. Gly) (Figure 2A,B).

**Figure 3:**
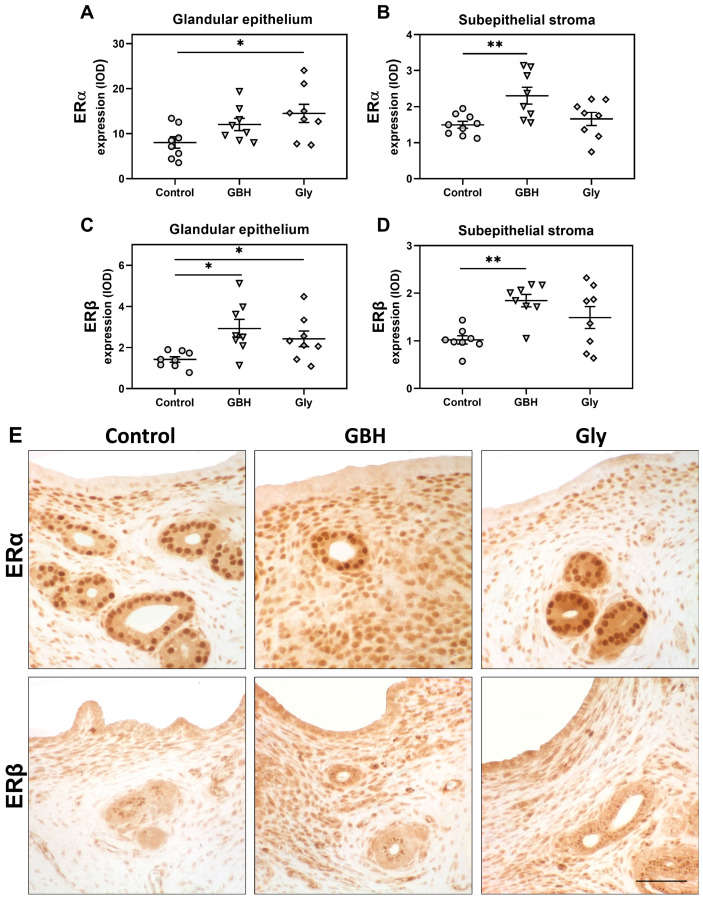
Effect of perinatal exposure to GBH or Gly on uterine expression of estrogen receptors (ERα and ERβ) on GD5. ERα quantification in (**A**) glandular epithelium and (**B**) subepithelial stroma. ERβ quantification in (**C**) glandular epithelium and (**D**) subepithelial stroma. The results of quantification are expressed as integrated optical density (IOD). Dots represent individual measures and solid lines represent the mean ± SEM of 8 animals per group. Asterisk denotes statistical significance (* *p* < 0.05; ** *p* < 0.01). (**E**) Representative photomicrographs of immunohistochemical detection of ERα and ERβ on uterine sections. Scale bar: 100 µm.

As shown in Figure 3, the protein expression of both ERs was also increased. ERα expression was increased in the subepithelial stroma of GBH animals (** *p* < 0.01) and in the glandular epithelium of Gly animals (* *p* < 0.05). On the other hand, ERβ expression was higher in the GBH group than in the control in both compartments analyzed (subepithelial stroma ** *p* < 0.01 and glandular epithelium * *p* < 0.05), but only in the glandular epithelium (* *p* < 0.05) of Gly animals.

**Figure 4:**
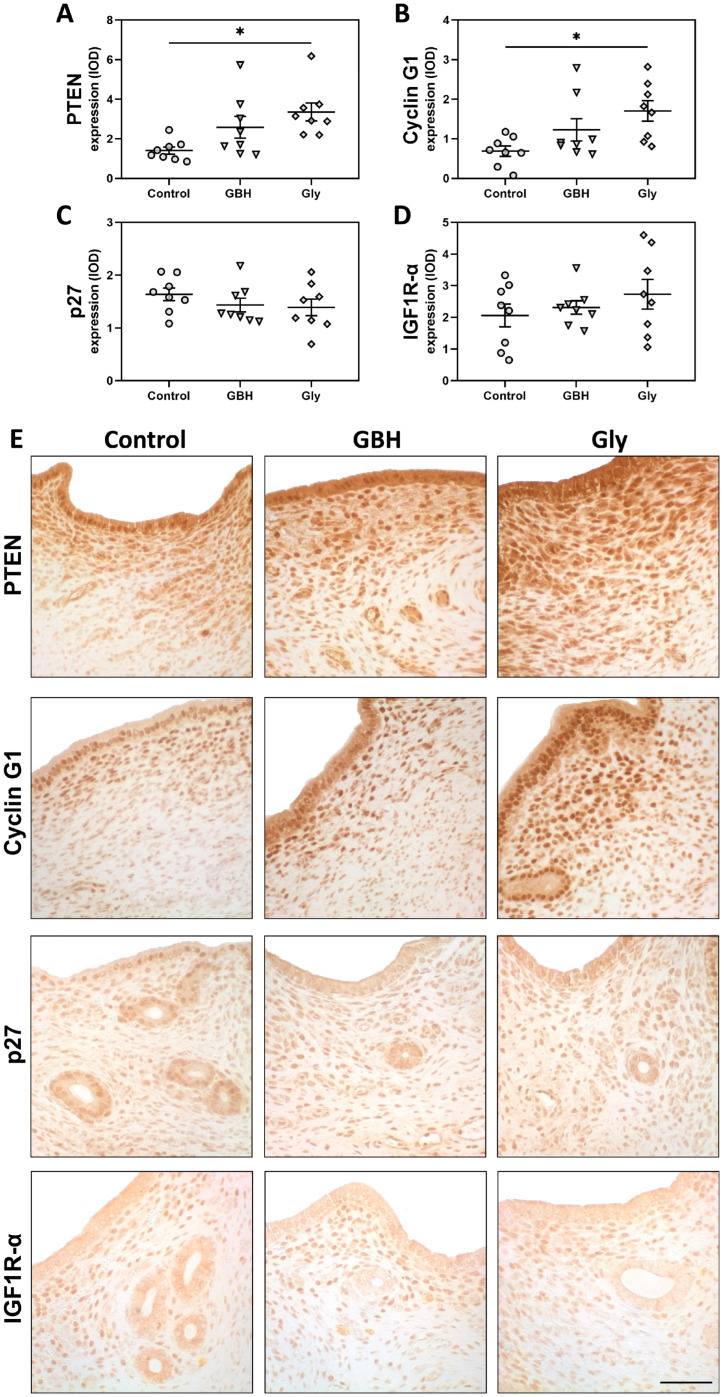
Effect of perinatal exposure to GBH or Gly on uterine expression of cell cycle regulators on GD5. Subepithelial stromal expression of (**A**) PTEN, (**B**) Cyclin G1, (**C**) p27, and (**D**) IGF1R-α. The results of quantification are expressed as integrated optical density (IOD). Dots represent individual measures and solid lines represent the mean ± SEM of 8 animals per group. Asterisk denotes statistical significance (* *p* < 0.05). (**E**) Representative photomicrographs of immunohistochemical detection of cell cycle regulators on uterine sections. Scale bar: 100 µm.

Regarding the expression of cell cycle regulators, increased levels of PTEN (* *p* < 0.05) (Figure 4A,E) and cyclin G1 (* *p* < 0.05) were detected (Figure 4B,E) in the subepithelial stroma of Gly-exposed rats compared to the control group. Although a similar tendency was observed for the GBH group, no significant differences were found. Protein expression of p27 and IGF1R-α was not different between groups (Figure 4C–E).

### 3.3. Protein Expression of a Key Endocrine Pathway for Endometrial Receptivity

**Figure 5:**
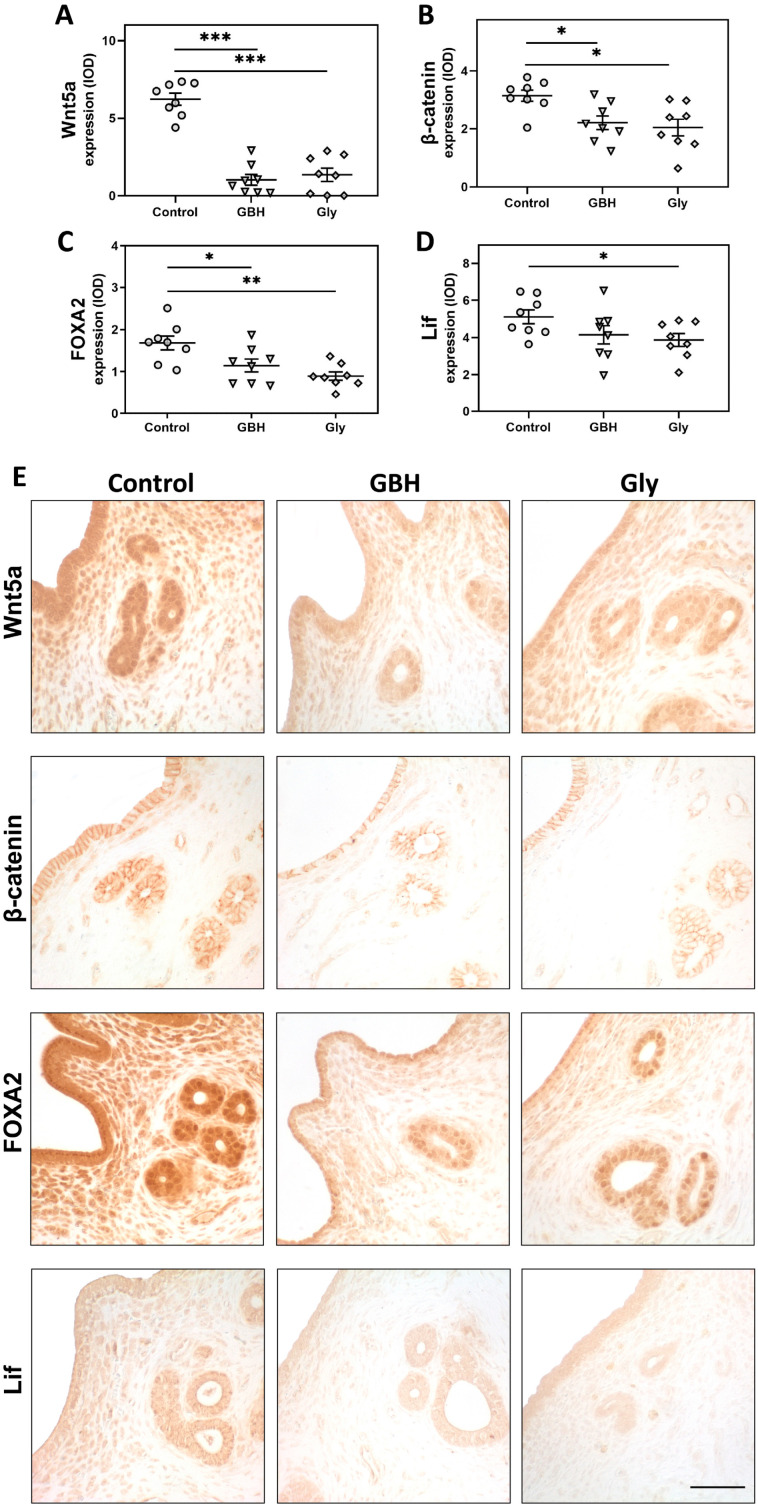
Effect of perinatal exposure to GBH or Gly on uterine expression of a key endocrine pathway for endometrial receptivity on GD5. Glandular epithelium expression of (**A**) Wnt5a, (**B**) β-catenin, (**C**) FOXA2, and (**D**) Lif. The results of quantification are expressed as integrated optical density (IOD). Dots represent individual measures and solid lines represent the mean ± SEM of 8 animals per group. Asterisk denotes statistical significance (* *p* < 0.05; ** *p* < 0.01; *** *p* < 0.001). (**E**) Representative photomicrographs of immunohistochemical detection of mediators of the endocrine pathway on uterine sections. Scale bar: 100 µm.

Both GBH and Gly decreased the protein expression of Wnt5a (*** *p* < 0.001), β-catenin (* *p* < 0.05), and FOXA2 (* *p* < 0.05 vs. GBH and ** *p* < 0.01 vs. Gly) in the uterine glandular epithelium compared to the control group (Figure 5A–C,E). Regarding Lif, a decreased expression was observed in the glandular epithelium of Gly-exposed animals (* *p* < 0.05) compared to the control group (Figure 5D,E). A similar trend was observed for the GBH group, but no significant differences were found (Figure 5D,E).

### 3.4. Colocalization of Wnt5a/β-Catenin and FOXA2/Lif

**Figure 6:**
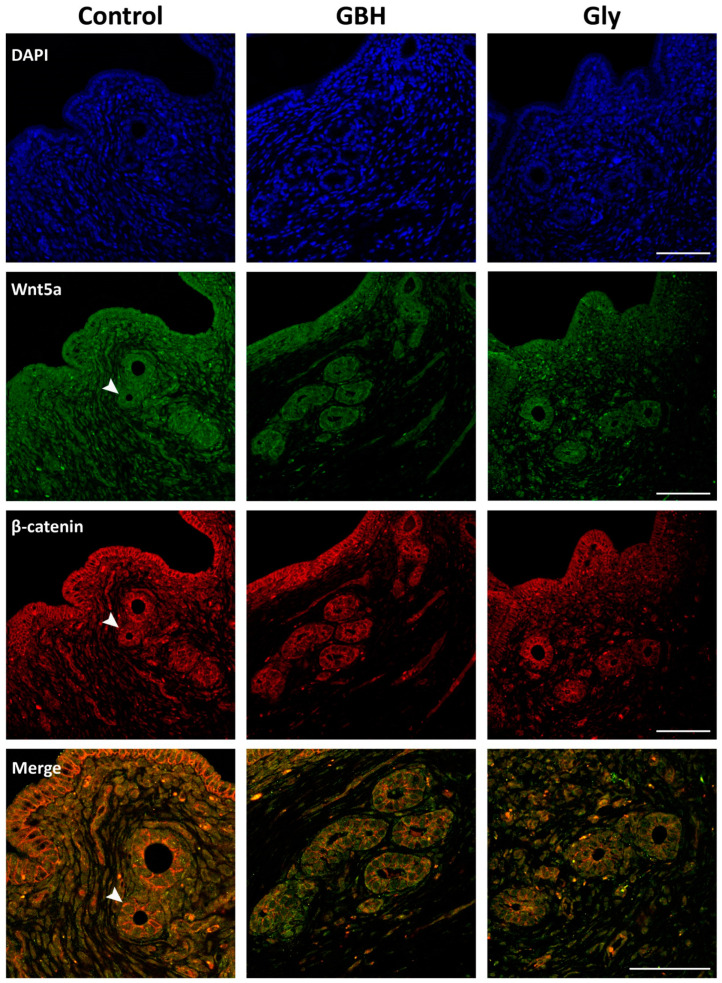
Representative photomicrographs of dual immunofluorescence staining for Wnt5a/β-catenin in the uterus of Control, GBH- and Gly-exposed rats on GD5. Images of DAPI nuclear staining (blue channel), Wnt5a (green channel), and β-catenin (red channel) makers were captured by confocal microscopy. The staining of each protein expression and its colocalization are indicated by white arrowheads. The original magnification was 40×, and the merge was 60×. Scale bar: 100 µm.

**Figure 7:**
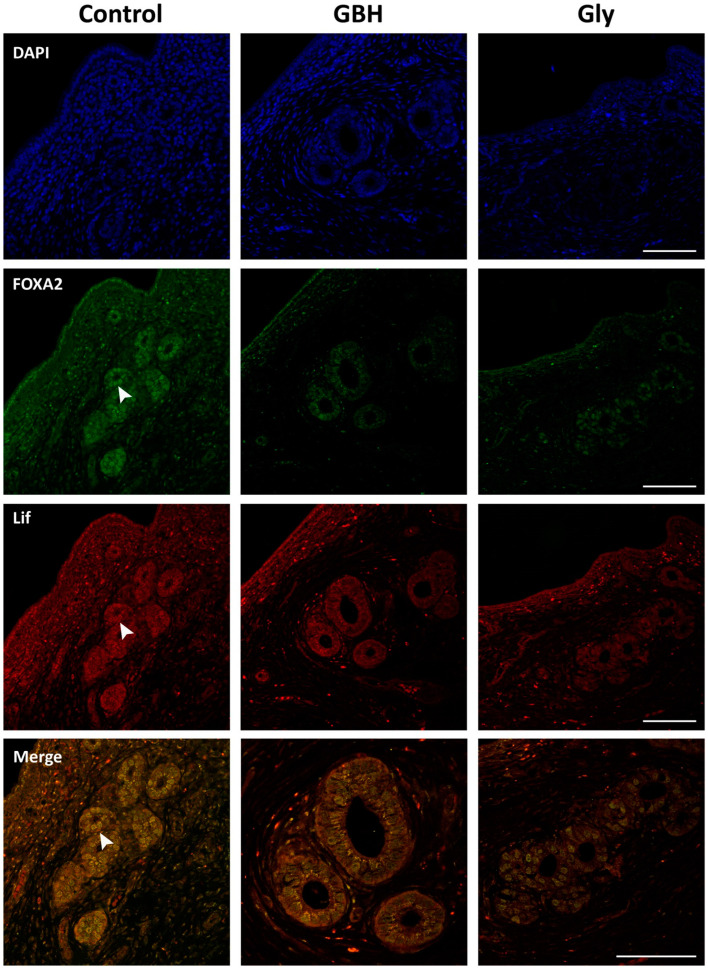
Representative photomicrographs of dual immunofluorescence staining for FOXA2/Lif in the uterus of Control, GBH-, and Gly-exposed rats on GD5. Images of DAPI nuclear staining (blue channel), FOXA2 (green channel), and Lif (red channel) markers were captured by confocal microscopy. The staining of each protein expression and its colocalization are indicated by white arrowheads. The original magnification was 40×, and the merge was 60×. Scale bar: 100 µm.

The colocalization patterns of Wnt5a/β-catenin and FOXA2/Lif in the preimplantation uterus were analyzed by a qualitative assessment of a dual immunofluorescence staining method. Figure 6 shows the coexpression of Wnt5a/β-catenin in the glandular epithelium, as demonstrated by merged images. While β-catenin was found to localize in the cell membrane, Wnt5a was found to have a cytoplasmic localization. Both molecules exhibited lower immunofluorescence in the GBH and Gly groups than in the control group, which is consistent with the immunohistochemical results. Figure 7 illustrates the coexpression of FOXA2/Lif in the glandular epithelium as merged images. The results of the dual immunofluorescence staining confirm the nuclear colocalization of FOXA2 and Lif in the glandular cells, as evidenced by the presence of yellow nuclei in the merged images of the control group. As with the immunohistochemical staining, a lower immunofluorescence intensity was observed for FOXA2 and Lif proteins in the Gly group, whereas the GBH group showed reduced immunofluorescence staining of FOXA2.

## 4. Discussion

Previously, we demonstrated that perinatal exposure to GBH or Gly at doses similar to those used in this study, impaired fertility in female rats [26] by increasing the preimplantation embryo losses [28]. We also found hormonal imbalance and uterine disruption of implantation-related genes during the preimplantation period, which could prematurely close the implantation window and cause the uterus to become refractory [26]. In the present study, we aimed to elucidate other potential mechanisms involved in the adverse effects of GBH and Gly by analyzing morphological, cellular, and molecular events for proper endometrial receptivity. Our results showed that rats perinatally exposed to GBH or Gly exhibited increased stromal proliferation associated with increased expression of ERs. Aberrant proliferation was also associated with dysregulation of PTEN and cyclin G1 in Gly-exposed females. Moreover, GBH and Gly animals showed a reduced number of glands along with decreased expression of the Wnt5a/β-catenin/FOXA2/Lif signaling pathway in the glandular epithelium, which plays a major role in the implantation process.

During the preimplantation period, the uterus undergoes morphological changes that are required for achieving a receptive stage [37]. Among them, endometrial proliferation is essential for the implantation process [37]. This cellular event is primarily controlled by P4 and estrogen, acting through their cognate nuclear receptors [3]. Indeed, prior to implantation, P4 from the newly formed corpora lutea initiates the proliferation of stromal cells, which is further stimulated by a peak in estrogen secretion [37]. Our findings show that GBH and Gly exposure increased stromal cell proliferation during the preimplantation period, along with increased expression of both ERs (ERα and ERβ) in the subepithelial stroma and/or glandular epithelium. Although the histologic structure of the subepithelial stroma did not show significant changes, functional alterations at the cellular and molecular level could contribute to a hostile environment for endometrial receptivity. In a previous study, and using the same experimental design, we found that GBH and Gly increased the serum levels of E2, without changes in P4 [26]. It is known that precisely regulated estrogen levels and ERα expression are critical for establishing the window of uterine receptivity [38,39]. Ma et al. [38] reported that higher E2 concentrations may close the implantation window in advance, rendering the uterus refractory. Other work using a conditional KO mouse of Ncoa6 (a regulator of estrogen sensitivity and signaling) showed that accumulation of ERα at the implantation site renders the uterus non-receptive with pregnancy failure [40]. Unlike ERα, studies using ERβ KO mice have shown that it is not essential for endometrial receptivity [41] but plays a protective role against the trophic effects of ERα [42]. Therefore, we propose that increased ERβ expression may occur to prevent the undesirable effects of ERα-mediated E2 in the context of cell proliferation. Taken together, our current and previous findings suggest that the high estrogen load and concomitant stromal proliferation may lead to a defective uterine environment that interferes with the transition to a receptive stage.

Consistent with the increased proliferation, we observed dysregulation of PTEN and cyclin G1 in the stroma of Gly-exposed rats. Although a similar trend was observed for GBH, no significant differences were found. PTEN, a proapoptotic phosphatase, negatively regulates the PI3K/Akt signaling, promoting cell cycle arrest [43]. Cyclin G1 can also inhibit cell cycle progression by interacting with regulators such as p53 tumor suppressor [44]. The fact that we found increased uterine expression levels of PTEN and cyclin G1 may reflect compensatory mechanisms attempting to counteract the excessive proliferation triggered by ERα overexpression. Both PTEN and cyclin G1 have been shown to play a pivotal role in endometrial receptivity and successful implantation in human and animal studies [45,46,47,48]. Cyclin G1 was found to be expressed in a spatial-temporal manner in the endometrium, reaching a peak at mid-late secretory phase, highlighting its importance for endometrial receptivity [49]. As for PTEN, its aberrant expression was associated with increased cell proliferation in the mid-secretory endometrium of infertile women with intramural uterine fibroids [50]. Moreover, transgenic mice that lack the PTEN gene in myometrial and stromal/decidual cells have demonstrated altered implantation and/or uterine glandular function, which contributes to infertility and fetal loss [51]. Interestingly, a link between ERβ and PTEN expression in controlling cell proliferation has been proposed. In accordance with our findings, ERβ can upregulate PTEN while inhibiting Akt, thereby preventing excessive proliferation [52]. Taken together, the imbalance in the levels of ER isoforms and cell cycle regulators reflects an altered endometrial proliferation, which may affect uterine receptivity.

Successful implantation also depends on adequate endometrial gland development and function. Endometrial glands provide cytokines and growth factors necessary for the assessment of uterine receptivity, stromal cell decidualization, and blastocyst implantation [53]. In the present work, we detected a reduced number of glands in the uterus of GBH- and Gly-exposed females during the preimplantation period. Similar findings were found by Almeida et al. [54] when evaluating the direct effect of a GBH on the uterus of F0 dams. The authors reported that early gestational exposure to a sub-lethal dose of Roundup^®^ (500 mg/kg) in rats increased preimplantation embryo losses, which was associated with a reduction in glandular epithelial cell height and uterine gland diameter at GD7 [54]. In our work, the low number of glands was accompanied by a decreased glandular expression of Wnt5a, β-catenin, and FOXA2 in both GBH and Gly animals and decreased glandular expression of Lif in the Gly group. In this signaling pathway, the FOXA2 transcription factor acts downstream of Wnt/β-catenin to regulate Lif expression, which is recognized as a key biomarker of endometrial receptivity [6,55]. This signaling pathway plays a critical role in regulating proper uterine gland formation and function, which are required for implantation [56]. In addition, it participates in the coordination of the crosstalk between the blastocyst and the endometrium [57,58]. KO mice for these genes exhibit glandular abnormalities and implantation defects [8,58,59]. In previous studies, we showed the disruptive uterine effect of GBH on this pathway using different experimental designs and animal models [34,60]. Exposure to a 2 mg of Gly/kg/day dose of a GBH during postnatal development in rats increased uterine sensitivity to an estrogen treatment and disrupted Wnt/β-catenin signaling [60]. In ewe lambs, we found that neonatal exposure to a GBH altered uterine development, associated with downregulation of Wnt5a, β-catenin, and FOXA2 protein expression [34]. Alterations in this endocrine pathway have been linked to altered pregnancy outcomes and various uterine pathologies, highlighting its important role in reproductive health. For example, clinical evidence shows that deficient expression of Lif is associated with the diagnosis of recurrent implantation failure or infertility without apparent cause in women [61]. Furthermore, decreased expression of Lif and FOXA2 has been observed in eutopic endometrial cells in patients with endometriosis [62,63,64], along with increased proliferative activity and migratory capacity [62]. It has been proposed that the lower expression of Lif observed in these patients may contribute to the difficulties experienced by women in achieving pregnancy [63]. All these findings suggest that GBH and Gly may alter glandular secretory activity during the preimplantation period, with negative consequences for endometrial receptivity and implantation success.

In summary, exposure to GBH and Gly results in alterations in endometrial proliferation and histomorphology, disruption of the Wnt5a/β-catenin/FOXA2/Lif signaling pathway, and aberrant expression of ERs and cell cycle regulators. Such alterations may lead to inadequate endometrial remodeling, ultimately interfering with the process of embryo implantation. Interestingly, GBH and Gly exposure elicited comparable effects, suggesting that the adverse outcomes are primarily driven by the active ingredient rather than other components present in the commercial formulation. This is consistent with our previous studies [26,28,65] and supports the fact that glyphosate herbicide may act as an EDC by inducing alterations in ERs expression and disrupting signal transduction in hormone-responsive cells [12,66].

Finally, the dose evaluated in this study provides insight into the mechanisms underlying the previously observed implantation failures associated with GBH or Gly exposure. Nevertheless, additional experiments are required to evaluate a broader range of doses and assess the dose-response relationship. Furthermore, it is necessary to ascertain whether both compounds affect the transcriptional activity of the molecules under evaluation. This is a pending task, and we are currently working on it, analyzing not only gene expression but also potential modifications in the epigenome.

## 5. Conclusions

This study demonstrates that perinatal exposure to environmentally relevant doses of GBH or Gly disrupts critical pathways involved in establishing endometrial receptivity during the preimplantation period in rats. Increased stromal proliferation coupled with altered ERs levels may create an abnormal uterine environment. Reduced gland number along with downregulation of the Wnt5a/β-catenin/FOXA2/Lif signaling pathway may contribute to gland dysfunction. Therefore, we propose that dysregulation of these interrelated processes that orchestrate endometrial remodeling for embryo implantation could underlay the previously observed implantation failure after GBH and Gly exposure. The comparable effects of GBH and Gly suggest that the active ingredient is a major contributor to reproductive toxicity. Our previous and current findings provide plausible mechanisms by which developmental exposure to this widely used herbicide could affect female fertility later in life by disrupting the intricate hormone-regulated processes that govern uterine receptivity.

## Figures and Tables

**Table 1:** Antibodies used for immunohistochemistry.

Antibodies	Dilution	Supplier
**Primary**		
Anti-Ki67 (clone MIB-5)	1/15	Dako Crop. (Carpinteria, CA, USA)
Anti-FOXA2	1/800	Generated and validated in our Institute [32]
Anti-Wnt5a	1/800	Generated and validated in our Institute [33]
Anti-Lif (sc-515931)	1/50	Santa Cruz Biotechnology Inc. (Santa Cruz, CA, USA)
Anti-β-catenin (sc-7963)	1/800	Santa Cruz Biotechnology Inc. (Santa Cruz, CA, USA)
Anti-PTEN	1/750	Generated and validated in our Institute [34]
Anti-Cyclin G1 (sc-7865)	1/25	Santa Cruz Biotechnology Inc. (Santa Cruz, CA, USA)
Anti-p27 (sc-528)	1/800	Santa Cruz Biotechnology Inc. (Santa Cruz, CA, USA)
Anti-IGF1R-α (sc-712)	1/100	Santa Cruz Biotechnology Inc. (Santa Cruz, CA, USA)
Anti-ERα (clone 6F-11)	1/100	Novocastra (Newcastle upon Tyne, UK)
Anti-ERβ (51-7900)	1/200	Zymed (San Francisco, CA, USA)
**Secondary**		
Anti-mouse (B8774)	1/100	Sigma-Aldrich (St. Louis, MO, USA)
Anti-rabbit (B8895)	1/200	Sigma-Aldrich (St. Louis, MO, USA)
Alexa Fluor 488 goat anti-rabbit (A-11034)	1/100	Invitrogen Molecular Probes (Eugene, OR, USA)
TRITC-conjugated goat anti-mouse (115-025-003)	1/100	Jackson Immunoresearch (West Grove, PA, USA)

FOXA2, forkhead box A2; Wnt5a, wingless-type MMTV integration site 5a; Lif, leukemia inhibitory factor; PTEN, phosphatase and tensin homolog; IGF1R-α, insulin-like growth factor 1 receptor α; ERα, estrogen receptor α; ERβ, estrogen receptor β.

## Data Availability

Data will be made available on request.

## Abbreviations

AMPA	aminomethylphosphonic acid
DAPI	4′6-diamidino-2-phenylindole dihydrochloride
E2	17β-estradiol
EDC	endocrine-disrupting chemical
EFSA	European Food Safety Authority
EPA	Environmental Protection Agency
ER	estrogen receptor
FOXA2	forkhead box A2
GBH	glyphosate-based herbicide
GD	gestational day
Gly	glyphosate
IGF1R-α	insulin-like growth factor 1 receptor alpha
IOD	integrated optical density
KO	knockout
LECH	luminal epithelial cell height
Lif	leukemia inhibitory factor
P4	progesterone
PND	postnatal day
PTEN	phosphatase and tensin homolog
Wnt5a	wingless-type MMTV integration site 5a.
